# Tracking slow modulations in synaptic gain using dynamic causal modelling: Validation in epilepsy

**DOI:** 10.1016/j.neuroimage.2014.12.007

**Published:** 2015-02-15

**Authors:** Margarita Papadopoulou, Marco Leite, Pieter van Mierlo, Kristl Vonck, Louis Lemieux, Karl Friston, Daniele Marinazzo

**Affiliations:** a Department of Data-analysis, University of Ghent, B9000 Ghent, Belgium; b UCL Institute of Neurology, Department of Clinical and Experimental Epilepsy, WC1N 3BG London, UK; c Institute of systems and robotics, Instituto Superior Técnico, Universidade de Lisboa, 1049-001, Portugal; d Medical Image and Signal Processing Group, Department of Electronics and Information Systems, Ghent University-IBBT, B9000 Ghent, Belgium; e Laboratory for Clinical and Experimental Neurophysiology, Ghent University Hospital, Ghent B9000, Belgium; f The Wellcome Trust Centre for Neuroimaging, University College London, Queen Square, WC1N 3BG London, UK

**Keywords:** DCM, dynamical causal modelling, SOZ, seizure onset zone, EEG, electroencephalography, CSD, cross spectral density, Dynamical causal modelling, Neural mass models, Seizure onset, Dynamical connectivity, Electroencephalography, Epilepsy

## Abstract

In this work we propose a proof of principle that dynamic causal modelling can identify plausible mechanisms at the synaptic level underlying brain state changes over a timescale of seconds. As a benchmark example for validation we used intracranial electroencephalographic signals in a human subject. These data were used to infer the (effective connectivity) architecture of synaptic connections among neural populations assumed to generate seizure activity. Dynamic causal modelling allowed us to quantify empirical changes in spectral activity in terms of a trajectory in parameter space — identifying key synaptic parameters or connections that cause observed signals. Using recordings from three seizures in one patient, we considered a network of two sources (within and just outside the putative ictal zone). Bayesian model selection was used to identify the intrinsic (within-source) and extrinsic (between-source) connectivity. Having established the underlying architecture, we were able to track the evolution of key connectivity parameters (e.g., inhibitory connections to superficial pyramidal cells) and test specific hypotheses about the synaptic mechanisms involved in ictogenesis. Our key finding was that intrinsic synaptic changes were sufficient to explain seizure onset, where these changes showed dissociable time courses over several seconds. Crucially, these changes spoke to an increase in the sensitivity of principal cells to intrinsic inhibitory afferents and a transient loss of excitatory–inhibitory balance.

## Highlights

• We propose a framework to characterise slow dynamical changes in the brain.
• Dynamical causal modelling finds the most likely connectivity among two brain areas.
• The synaptic weights defining these connections are tracked in time.
• We analyse brain activity of an epileptic subject, at the focus and just outside it.
• We point to modulations of synaptic connections as responsible of the seizure.

## Introduction

In this paper we test the hypothesis that systematic changes in observed cross spectral density of electroencephalographic signals can be explained in terms of fluctuations in key model parameters (such as the strength of recurrent inhibitory connections to specific neuronal populations) — and that slow fluctuations in one or more of these parameters can explain changes in brain activity. The methodological advance included here is the use of dynamic causal modelling (DCM) to provide biophysically informed characterisations of electrophysiological responses in terms of slow changes in synaptic efficacy. DCM is a Bayesian framework for comparing different hypotheses or network models of observed (neurophysiological) time series.

Although DCM has been validated in the context of event related responses (Garrido et al., 2009) and steady-state or induced responses (Moran et al., 2011a), it has not been used to track short-term fluctuations in synaptic efficacy. Our focus is therefore on the validity of DCM in recovering slow (pathophysiological) changes in synaptic connectivity from electrophysiological time series. We first establish face validity using physiologically realistic simulations (using the same model used to characterise our empirical data) and then apply the same procedure to real data, intracranial electroencephalography signals from an epileptic subject. This shows that DCM provides veridical estimates of how the data were generated and establishes the identifiability of the model used for subsequent empirical analyses. The empirical application provides a proof of principle that changes in synaptic efficacy can be measured at single subject level — and shows that pathophysiological changes beyond the seizure onset zone are necessary to explain seizure activity.

We chose epileptic seizure onset as a validation of this framework given the nature of the brain dynamics in this pathological condition. In patients affected by drug-resistant epilepsy and for which surgical treatment is thus sought, intracranial EEG is considered the gold standard for delineating the seizure onset zone (SOZ). Intracranial recordings allow one to characterise seizure activity with a high temporal resolution and track its temporal evolution. It should be noted that the onset of seizure activity may not be limited to the seizure onset zone but may be modulated – or be mediated by – distributed dynamics in brain networks.

The need to accurately track and quantify seizure dynamics has led to the development of multivariate time series analyses of signals recorded simultaneously (Pereda et al., 2005; Lehnertz, 1999). The fact that brain function involves distributed neuronal activity – and that this functional integration is modulated by cognitive or pathophysiological factors – motivates a focus on dynamical interactions not limited to the seizure onset zone but involving distal regions. Consequently, methods grounded in information theory and dynamical systems represent promising candidates, given their potential to describe the intricate pattern of dependencies in multivariate time series.

## Materials and methods

This report introduces the concepts and procedures that allow one to estimate slow changes in synaptic parameters that may underlie changes of brain states. Its focus is on describing the approach and providing some face validation (showing it does what it says it does). This validation uses data from a single patient to provide plausible model architectures and parameters – that were used to create synthetic data. We then invert models of those data – to ensure we can recover the (known) parameters. In subsequent publications we will apply this analysis to examine its reproducibility and predictive validity in patient cohorts.

We used data recorded from a patient (female, 50 years old) with refractory epilepsy who had a total of three epileptic seizures during video-EEG monitoring. The patient was implanted at Ghent's University Hospital with 52 intracranial contacts monitoring eight regions of interest according to the following configuration: bilateral occipito-hippocampal depth electrodes with 12 contacts each (Left: LH1–LH12, Right: RH1–RH12); four subdural strips with four contacts each, monitoring the anterior temporo-basal and the posterior temporo-basal region (Left: anterior LTA 1–LTA4 and posterior LTM1–LTM4, Right: anterior RTA1–RTA4 and posterior RTM1–RTM4) and two subdural strips of six contacts each, monitoring the temporo-lateral region (Left: LTP1–LTP6, Right: RTP1–RTP6). Based on the invasive video-EEG monitoring the ictal onset zone was localized to the left hippocampus, primarily involving LH2–4. The patient underwent a selective amygdalo-hippocampectomy in 2007 and has been seizure free since that time.

The data were epoched to a segment starting 20 s before electroencephalographic seizure onset (pre-ictal). The segment included the whole duration of seizure activity, which varied over the three seizures from 229 to 262 s. The beginning and the end of the seizure were marked by epileptologists. The sampling frequency of the EEG recordings was 256 Hz and a band pass filter was applied to the data (0.5 Hz–48 Hz). The intracranial data were re-referenced by applying a bipolar montage corresponding to a series of overlapping bipolar derivations (acting as spatial filter).

**Fig. 1:**
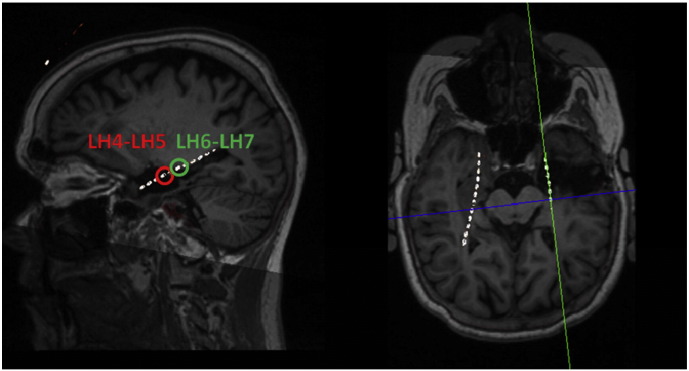
Location of the two intracranial electrodes and sources considered in the dynamic causal modelling. The stereotactic trajectories of the electrodes are superimposed upon the individual structural MRI scan. The leftmost circle (LH4–LH5) corresponds to the first source — considered the onset zone, while the one on the right (LH6–LH7) indicates our second source.

Our analysis focused on two sources of activity: a primary source within the subsequently resected area, whose activity was confirmed to be part of the seizure onset zone after postsurgical follow-up (LH4–LH5) and a second source (LH6–LH7) lying just outside the area of resection (Fig. 1). 10 s of activity before and after seizure onset were modelled, where each segment was partitioned into nine contiguous windows with 50% (1 s) overlap, for a total of 18 time windows.

### Dynamic causal modelling

Dynamic causal modelling (DCM) is an established procedure in the analysis of functional magnetic resonance imaging in brain mapping (Daunizeau et al., 2011; Friston et al., 2012) and is now being used increasingly for the characterisation of electrophysiological time series. DCM is used to identify the connectivity architectures and connection strengths in distributed networks using (observable) measurements of (hidden) neuronal activity. It is essentially a Bayesian model comparison scheme that allows one to evaluate competing hypotheses (or architectures) in terms of their Bayesian model evidence or marginal likelihood. Having established the best model architecture, Bayesian estimates of the model parameters provide a quantitative characterisation of effective connectivity and other (synaptic) parameters. There is an extensive literature on the validation of DCM ranging from face validation studies (David et al., 2006) to validation in terms of multimodal measurements (David et al., 2008a), pharmacological manipulations (Moran et al., 2011a, 2011b) and psychophysical constructs (Brown and Friston, 2012). Its predictive validity has been established in a number of studies in terms of pathophysiology (Boly et al., 2011).

Quantifying the effective connectivity between coupled neuronal sources corresponds to inferring the causal relationships among them, in relation to a model of those dependencies (Stephan et al., 2007). The nodes of dynamical causal models can reflect different regions in the brain that are connected by (extrinsic) forward and backward connections according to the laminar specificity established by Felleman and Van Essen (1991). Different models can be used within DCM depending on the question of interest and the most informative data features at hand (Moran et al., 2013).

The analysis described in this section uses standard procedures developed in DCM for cross spectral density (CSD) (Friston et al., 2012), which is a generalisation of DCM for steady state responses. The CSD is the Fourier transform of the cross-correlation function, which summarizes the activity and statistical dependencies among channels in frequency space. It can be thought of as reporting the correlations at each frequency. Usually, DCM for CSD is applied to a single cross spectrum (for a given time series). However here, we model successive time windows; effectively summarizing the time series with its time–frequency decomposition. The reason that we choose these (cross spectral) data features is that they contain information about the underlying connectivity that can be accessed through estimating the spectral density (second-order statistics) of endogenous activity. This contrasts with modelling of the time series per se, which would require the time-dependent (first-order statistics) endogenous input (e.g., the input associated with a stimulus in the event related potential studies).

This DCM has been applied in several contexts previously. Technical details can be found in Moran et al. (2007, 2009) and its applications to in vivo synaptic assays are described in Moran et al. (2011a, 2011b). In brief, parameter estimation uses standard (variational) Bayesian model inversion, where the forward or generative model predicts cross spectral responses from models of coupled neuronal masses. These models are specified in terms of equations of motion (i.e., state space models in continuous time). The equations are based upon standard neural mass models and define transfer functions linking endogenous activity at each source to spectral responses measured over channels. This allows one to predict observed cross spectra for any given model architecture and parameters; thereby providing an observation or forward model of spectral responses. Inversion of this model provides the model evidence (for model comparison) and posterior densities over model parameters in the usual way. Usually, one tries to explain differences in spectral responses among conditions, in terms of changes in a small number of synaptic parameters, where these changes define the model.

The novel aspect of the current analysis is the application of a standard DCM to test for slow changes in model parameters (e.g., the strength of inhibitory recurrent connections). We do this by exploiting the differences in timescales between the fast neuronal activities and slow changes in synaptic efficacy. This allows one to make local stationarity assumptions and treat successive epochs of data as different conditions — where these conditions or epochs induce fluctuations in specified parameters. Again, using the usual Bayesian model comparison procedures, we can then identify changes in parameters during seizure onset that best explain the sequence of (cross spectral) responses.

For this study, we employ a DCM for cross spectral densities (CSD) (Friston et al., 2012), which is a generalisation of DCM for steady state responses (Moran et al., 2007, 2009) to the complex domain. In brief, this form of DCM is used to explain complex cross spectral responses from multiple channels (here two channels) in terms of coupled sources, each comprising several neuronal populations or neural masses (here four neuronal populations). Given the parameters of a neural mass model, it is easy to compute the transfer functions that map from endogenous neuronal fluctuations within each source to the observed responses in channel space. These transfer functions specify the cross spectral densities one would expect to observe empirically. Effectively, the dynamic causal model is a forward model that includes the neuronal process generating neuronal states and the (electromagnetic) mapping from neuronal states to measured data. Bayesian model inversion is then used to estimate the parameters that best explain empirical spectra and provide the Bayesian model evidence for the particular model used (e.g., with or without changes in particular connections).

**Fig. 2:**
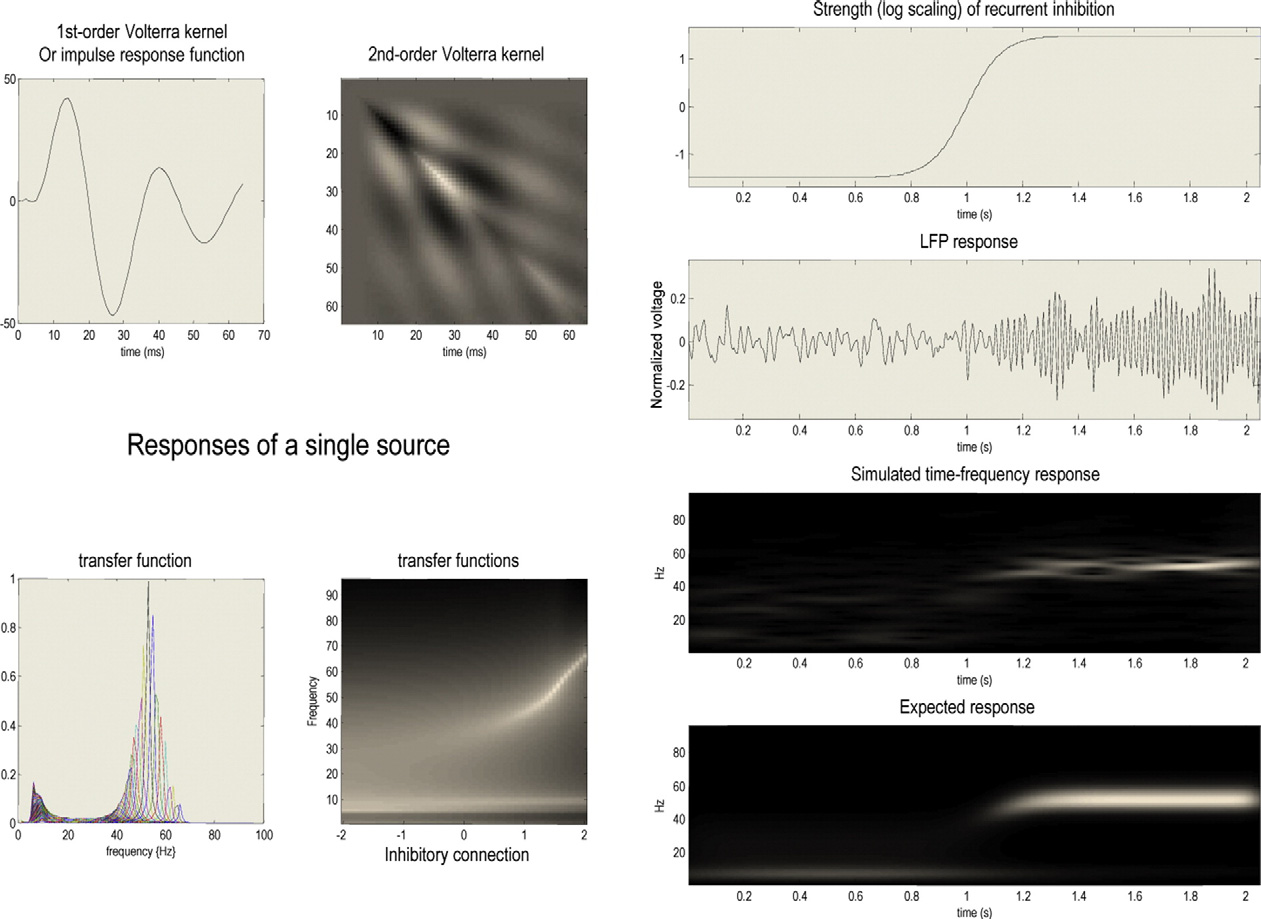
Left panels: Response characteristics of a single source within a dynamic causal model of the sort used in subsequent analyses (a canonical microcircuit neural mass model). The upper panels show the first and second order impulse response functions of time in terms of their impulse responses (Volterra kernels). These reflect the impact of inputs on observed responses and are a function of the model's parameters. The equivalent formulation of the impulse response in frequency space is shown in the lower panels graphically (on the lower left) and in image format for different values of the inhibitory connection (on the lower right). These are called (modulation) transfer functions and represent the frequencies in the inputs that are expressed in the output. In this example, we have shown the responses as a function of (the log scaling of) recurrent inhibitory connectivity to one of four neuronal populations comprising the source (see Fig. 3). These response functions can be used to compute the expected cross spectral density for any values of the parameters. Right panels: these illustrate changes in neuronal activity when increasing recurrent inhibition. The top panel shows strength of recurrent inhibition as a function of time in seconds, while the second panel shows a simulated response obtained by integrating the neural mass model with random fluctuating inputs, with the value of inhibitory connection set to 1.5. The simulated time frequency response is shown below in terms of the spectral power over 4 to 96 Hz. The lowest panel shows the predicted power based upon the transfer functions shown on the left.

In summary, DCM solves the inverse problem of recovering plausible parameters (of both neuronal dynamics and noise) that explain observed cross spectra. It uses standard variational Bayesian procedures (Friston et al., 2007) to fit time-series or cross spectra – under model complexity constraints – to provide maximum a posteriori estimates of the underlying model parameters and the evidence for any particular model; see Friston et al. (2012) for more details in this particular setting. Fig. 2 illustrates the basic idea behind the application of dynamic causal modelling to cross spectral responses. The key point made by this figure is that changes in connectivity can have profound effects on spectral behaviour responses to endogenous input. It is these effects that are used to estimate (changes in) the underlying connectivity (Friston, 2014). If we take the modifications in the amplitude and frequencies produced by changes in model parameters as a simple model of seizure onset, one can use the predicted spectral responses as a likelihood model of empirical responses and thereby estimate the time-dependent changes in parameters. The simulations reported in Fig. 2 can be reproduced using the seizure onset demonstration in the neuronal modelling toolbox of the academic SPM freeware (http://www.fil.ion.ucl.ac.uk/spm). These simulation results use standard parameter values (prior expectations: see Table 1).

In the analyses reported below, we modelled frequencies between 8 and 48 Hz, thereby removing fluctuations in the theta range and allowing the model to explain activity at higher frequencies before and after seizure onset. The choice of frequencies to model is partly dictated by the phenomenology of observed seizure activity and the level of modelling supported by the data. Clearly, seizure activity encompasses both low (e.g., theta) and high (gamma) frequencies — so why did we restrict the range? This choice was partly motivated by the level of detail in the models (i.e., complexity) supported by the data. In other words, to maximize model evidence, models should provide an accurate account of spectral responses but in a parsimonious way (see below). This places constraints on the range of frequencies that can be modelled (given a limited number of parameters that entail synaptic time constants that shape spectral responses). The neural mass model used in this paper was chosen to explain frequencies between alpha and (high and low) gamma. In this case, the most prominent seizure related changes were observed largely in the beta band.

### The neural mass model

Neural mass models comprise ordinary differential equations that (using a mean field approach) model the dynamical behaviour of neuronal populations. These models have been developed to accommodate interacting cell types and their connectivity (Moran et al., 2013). In this work we use the canonical microcircuit neural mass model (CMC) based on the extrinsic and intrinsic connectivity described in Bastos et al. (2012). This particular model has been used previously to characterise phenomena like intrinsic gain control mechanisms in hierarchical visual processing (Brown and Friston, 2012) to impaired top-down connectivity in minimally conscious states (Boly et al., 2011).

**Fig. 3:**
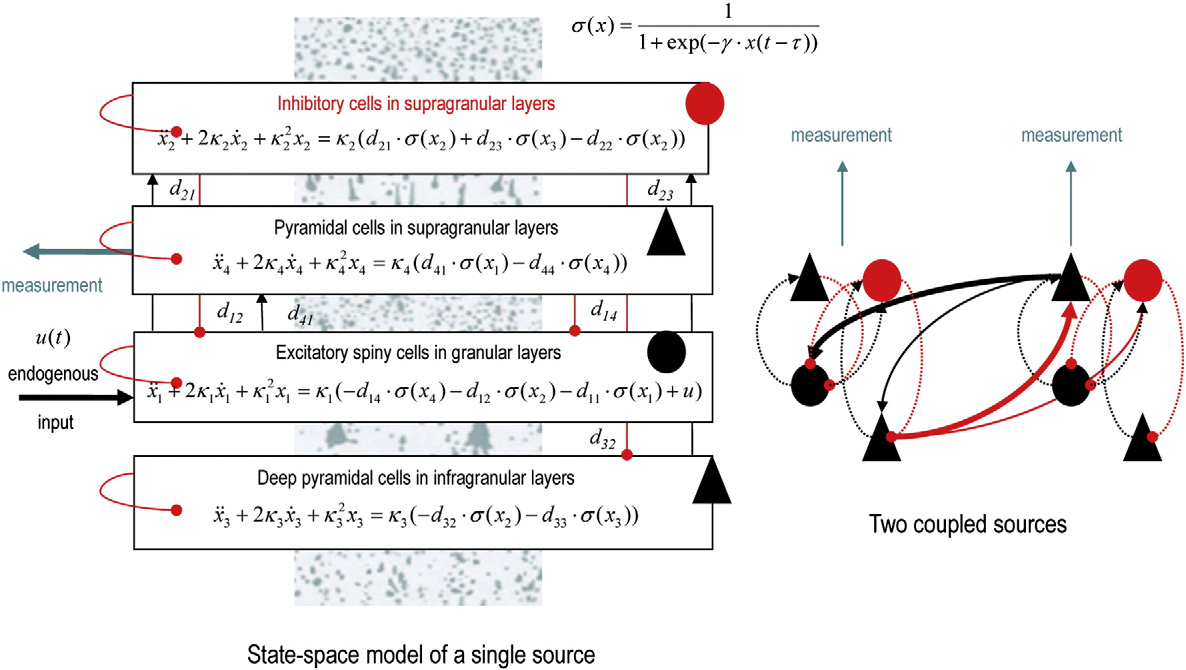
This schematic illustrates the state-space or dynamic causal model that we used for the dynamic causal modelling reported subsequent figures. Left panel: this shows the differential equations governing the evolution of depolarisation in four populations constituting a single electromagnetic source (of EEG, MEG or local field potential measurements). These populations are divided into input cells, inhibitory interneurons and (e.g., superficial and deep) principal cell populations that constitute the output populations. The equations of motion are based upon standard convolution models for synaptic transformations, while coupling among populations is mediated by a sigmoid function of (delayed) mean depolarisation. The slope of the sigmoid function corresponds to the intrinsic gain of each population. Intrinsic (within source) connections couple the different populations, while extrinsic connections couple populations from different sources. See Table 1 for a list of key parameters and a brief description. Right panel: this shows the simple two source architecture used in the current paper. The intrinsic connectivity (dotted lines) and extrinsic connectivity (solid lines) conform to the connectivity of the canonical microcircuit and the known laminar specificity of extrinsic connections (Bastos et al. 2012). Excitatory connections are in red and inhibitory connections are in black. Endogenous fluctuations drive the input cells and measurements are based on the depolarisation of superficial pyramidal cells.

The CMC model distinguishes between forward and backward connections that arise from different types of principal cells (e.g., superficial and deep pyramidal cells in the cortex). In addition, this model includes excitatory and inhibitory populations that send intrinsic connections to other populations (e.g., of excitatory spiny stellate and inhibitory interneurons in the cortex). Fig. 3 shows the architecture of the two source CMC model we used, with four populations per source and extrinsic connections between the sources. The boxes detail the equations of motion that constitute the neural mass model of a single source. These are delay differential equations because the sigmoid function of presynaptic input operates on the mean depolarisation of the presynaptic source in the recent past — to accommodate axonal conduction delays. Intrinsic conduction delays are about 1 ms while extrinsic delays are about 8 ms. This figure shows the four populations in relation to their laminar relationships in the cortex. Note that the equations of motion in the figure appear to violate Dale's principle of one transmitter per cell type; for example, they include inhibitory connections from excitatory populations. This reflects the complexity of neural mass models that can be supported by the data at hand. In short, for any given data there will be an optimal model evidence (or marginal likelihood) that can be decomposed into accuracy and complexity. This means that models have to have the optimal level of complexity (i.e., number of parameters) to maximize model evidence. In the context of the neural mass model used in this work, several inhibitory interneurons populations have been absorbed into a negative effective connectivity. For example, recurrent connections among superficial pyramidal cells are assumed to be mediated bi-synaptically by intervening inhibitory interneurons (that are not modelled). This reproduces the same dynamics but avoids using too many model parameters.

One might ask whether using a (cortical) canonical microcircuit model is appropriate for subcortical structures such as the hippocampus modelled in this paper. Strictly speaking, this is an issue that would be best addressed using Bayesian model comparison, for example comparing the canonical microcircuit with the bespoke model of hippocampal circuitry described in Moran et al. (2015). However, for our current purposes having four subpopulations appears to be sufficient. Our previous experience with these models suggests that the canonical microcircuit model is sufficient to model hippocampal responses; perhaps because the basic connectional architecture is conserved over the cortex and structures like the hippocampus (i.e., a circuit with excitatory input and output cells and an inhibitory and excitatory pair).

### Bayesian model comparison

**Fig. 4:**
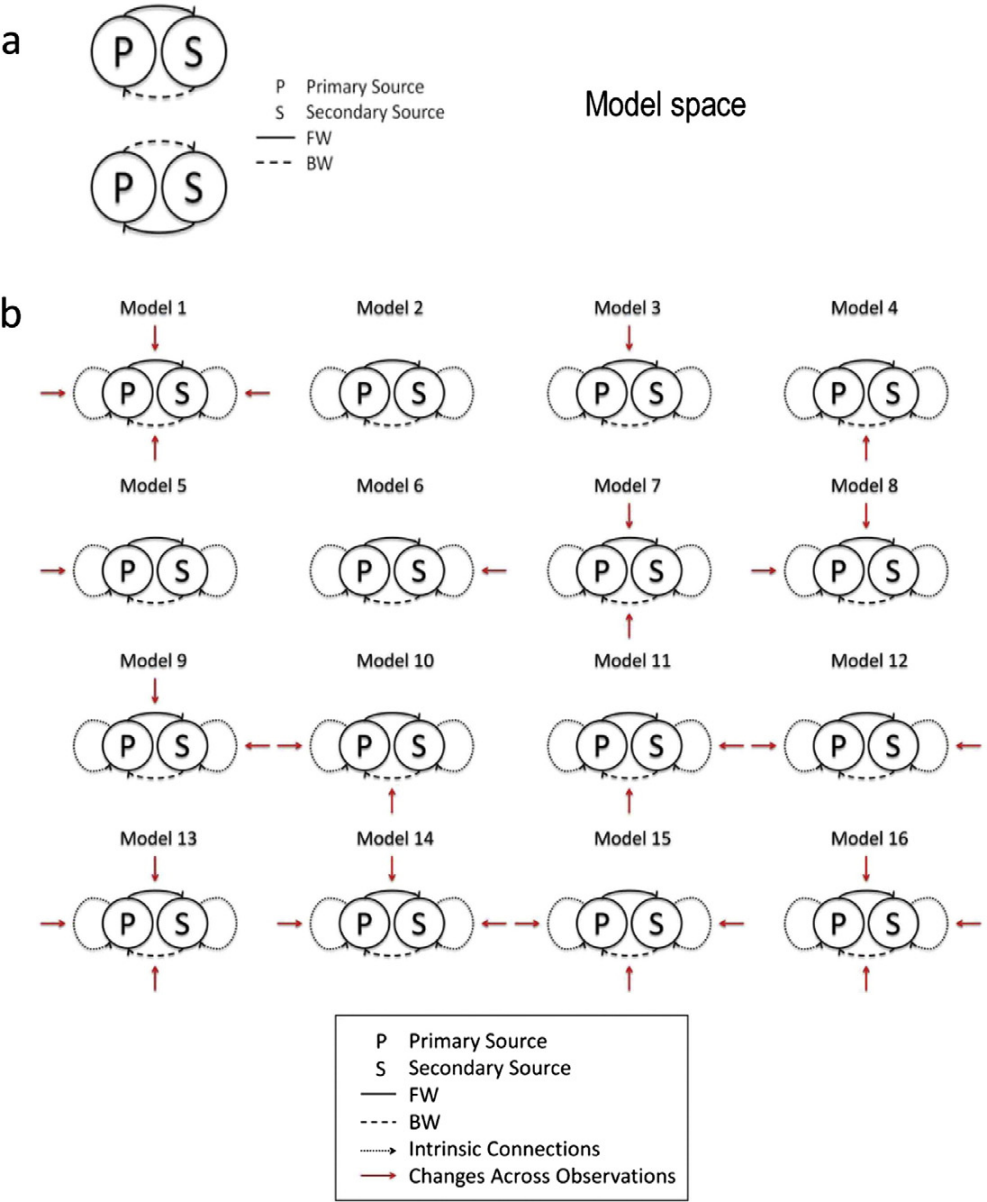
a) Alternative model architectures for the extrinsic coupling between the primary and secondary sources. FW: forward connectivity; BW: backward connectivity. b) Schematic showing the 16 models we tested. These models correspond to alternative hypotheses about changes in synaptic coupling that can explain changes in spectral activity before and after seizure onset. The 16 models correspond to all combinations of changes in intrinsic connectivity (in the primary and secondary sources) and changes in forward and backward extrinsic connections. The changes in intrinsic connectivity were modelled as changes in the inhibitory recurrent or self connections among superficial pyramidal cells.

DCM was used to compare alternative hypotheses about which synaptic parameters were responsible for changes in cross spectral density during seizure onset — after establishing the basic architecture of extrinsic connections between the two sources. Our analyses were therefore based upon a two-step Bayesian model comparison procedure. In the first step, we identified the best model architecture — distinguishing between extrinsic forward and backward connections between the primary ictal source (LH4–LH5) to the secondary source (LH6–LH7) and the reverse architecture with backward connections from the primary to the secondary source (Fig. 4a). To disambiguate these two architectures we inverted all 18 time windows, allowing only a number of connections to change over time (see below). The most likely architecture was identified using Bayesian model comparison by pooling the evidence for the two alternative models over windows from all three seizures. This allowed us to establish whether the extrinsic connections from the first to the second source were of a forward or backward type (and vice versa).

The second stage of the analysis focused on the changes in intrinsic and extrinsic connectivities over time windows — and implicitly between pre-ictal and ictal states. Using the most likely model from the first step, we allowed various combinations of intrinsic and extrinsic connections to change over time (using third order polynomial functions of time, for the pre- and post-ictal windows). This allowed us to estimate the trajectory of coupling parameters within and between pre-ictal and ictal time windows — while holding all other parameters at the same values (e.g., conduction delays that should not change over time). The parameters we allowed to vary corresponded to extrinsic connection strengths between the two sources and their intrinsic connectivity. Following Wendling et al. (2005) we associated changes in intrinsic connectivity with the influence of inhibitory interneurons on (superficial) principal cells. The possible combinations are described by 16 models, with and without changes in: intrinsic connectivity in the primary source, intrinsic connectivity in the secondary source, forward connectivity and backward connectivity. A schematic of the 16 models tested is provided in Fig. 4b. It is changes in these connections that we hoped would explain both variability within the pre- and ictal states and the slow changes that underlie seizure onset.

### Face validation studies

To establish the face validity of this application of DCM, we analysed both simulated and real data. Crucially, the parameters used to simulate the (cross spectral) data were based upon biologically plausible estimates from the empirical data. However, because the simulated data were generated under known model parameters (connectivity and time-dependent changes) we knew the ground truth and we could establish that the true values fall within the 90% posterior confidence intervals. For the simulation studies, we generated 18 time windows of cross spectral data using the prior expectations for intrinsic and extrinsic connectivities for the first (nine pre-ictal) windows and mono exponentially decaying connection strengths during the (nine) ictal windows. We used forward connections from the primary to the secondary source and restricted seizure-related changes in connectivity to the forward connectivity and intrinsic inhibitory connections to superficial principal cells in both sources. These changes modelled a transient increase in the excitability of principal cells mediated by both intrinsic and extrinsic connectivities. The time constant of extrinsic decay (back to the prior expectation) was 2 s and the time constant of intrinsic decay was 8 s. The values of all other parameters were set at the posterior estimates from the empirical analysis of the first seizure described below.

To create realistic simulated data, residuals from the empirical analyses (randomly permuted over windows) were added to the simulated cross spectra to ensure that the sampling noise and its correlation structure had the same amplitude and form that would be encountered empirically. We used a signal to noise ratio of four, over all channels and time windows.

### Analysis of real data

We performed model comparison and repeated the above analysis to estimate the trajectory of model parameters for the three successive seizures. These analyses used Bayesian updating, where the posteriors from the first seizure were used as priors for the second seizure and similarly for the second and third seizures. This enabled us to accumulate evidence for different models, while allowing for changes in parameters that could change from seizure to seizure (for example electrode gain). We then pooled the evidence over seizures to identify the best model. Finally, we identified the parameter estimates of the best model to quantify trajectories in the parameter space for each seizure.

## Results

### Face validation

**Fig. 5:**
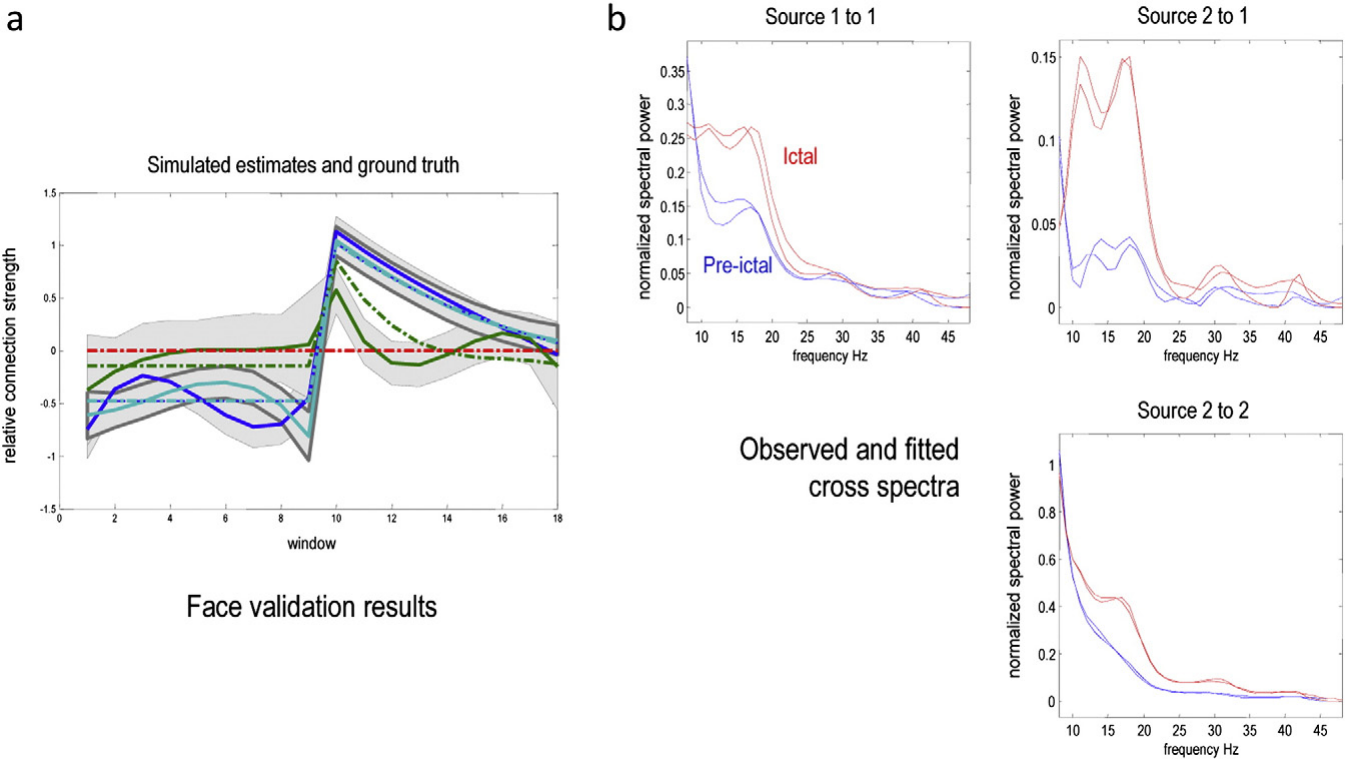
a). This panel shows the time-dependent changes in (log scaling of) the intrinsic and extrinsic connections as a function of window number. The posterior expectations correspond to the coloured lines (blue and cyan correspond to intrinsic connectivity, while green and red lines report the forward and backward connectivity respectively). The true values are shown as broken lines, the posterior estimates as full lines and the 90% confidence intervals as grey areas. b) Predicted (solid lines) and observed (dotted lines) cross spectra for pre-ictal (blue) and ictal (red) periods. This example uses average spectra from the first seizure to illustrate the quality of the model fit and the spectral data features that inform the posterior estimates of the model parameters. The absolute values of the (complex) cross spectra are shown in the upper right panel.

The results of the face validation (simulation) study are shown in Fig. 5a: this shows the time-dependent changes in (log scaling of) the intrinsic and extrinsic connections as a function of window number. The posterior expectations correspond to the coloured lines (blue and cyan correspond to intrinsic connectivity, while green and red lines report the forward and backward connectivities respectively). The true values are shown as broken lines and the posterior estimates as full lines. In this example, we precluded changes in the backward connections from first to the second source. There is a pleasing correspondence between the posterior estimates and the true values. Indeed, for the intrinsic changes (blue and cyan) they are virtually indistinguishable. Note the characteristic overconfidence of these estimators (due to the mean field approximation in the variational scheme). This means that in some cases the true value lies just outside the 90% confidence intervals (grey areas). This is particularly evident for the forward connectivity (green) shortly after seizure onset. These results suggest that the trajectory of parameters can be recovered even under fairly realistic levels of sampling noise and biologically plausible values for the neuronal dynamics.

### Empirical analyses

A typical model fit to the observed (empirical) cross spectra is provided in Fig. 5b — showing the characteristic changes in complex cross spectra from a pre (blue) to post (red) ictal window. This example shows the typical excess of power (and coherence) in the beta band following seizure onset. Bayesian model comparison of competing models with different extrinsic (forward and backward) connections suggested that we can be almost certain that the forward connection originates in the primary source, with a log evidence difference of over 100 (Penny et al., 2004). Differences in log evidence are the same as log Bayes factors, where the Bayes factor is an odds ratio comparing the evidence or marginal likelihood of two models.

**Fig. 6:**
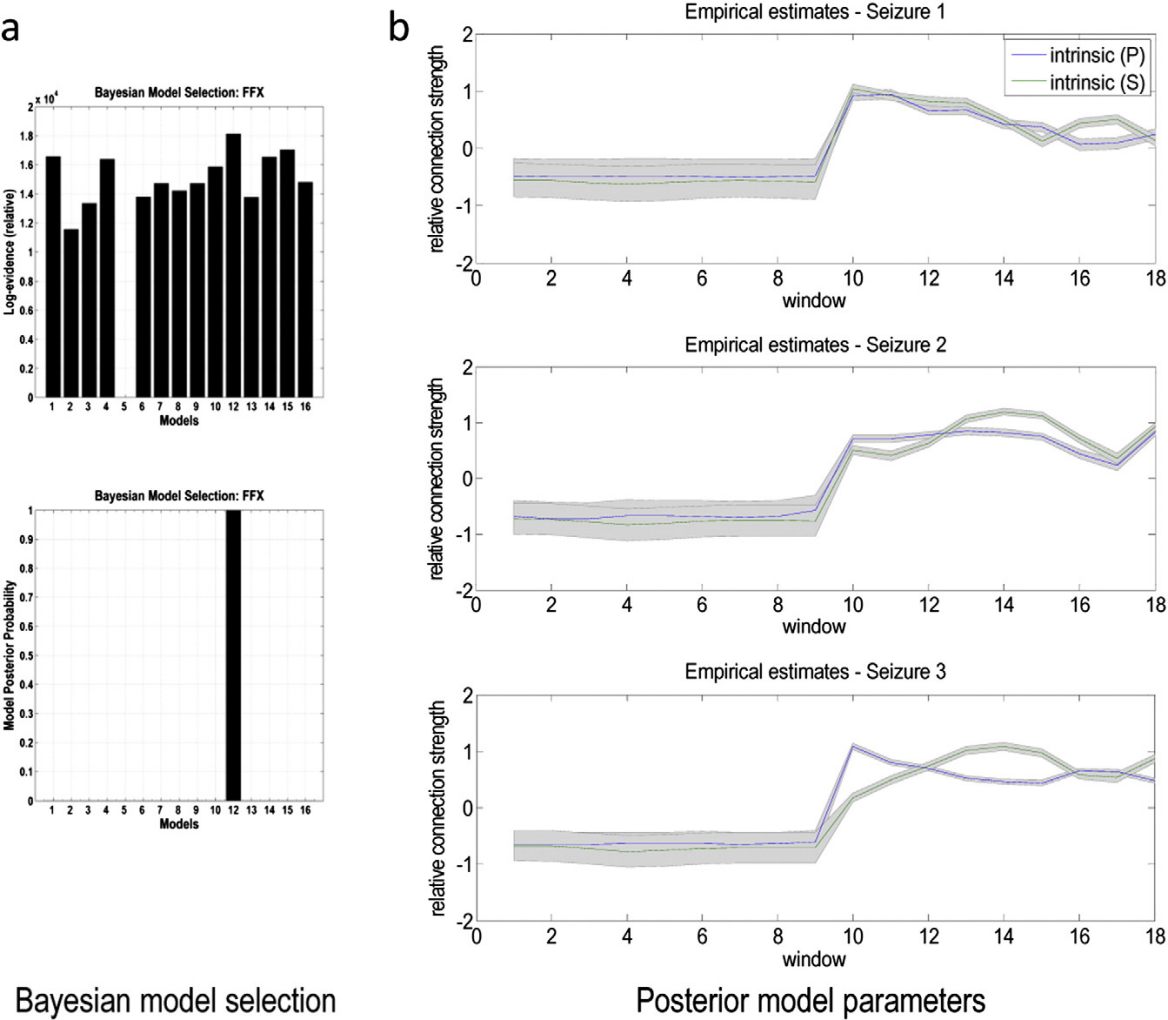
a) Upper panel: these are the variational free energy approximations to log model evidence for the 15 models covering changes in one or more synaptic parameters before and after seizure onset. Lower panel: this shows the corresponding posterior probability over models and identifies a single model with almost 100% posterior confidence. b) Changes (across consecutive windows, for each of the three seizures) in the synaptic parameters that were allowed to change in the winning model. Changes are shown in terms of log scaling to clarify the profile of changes over time. Each window corresponds to 1 s. The blue and the green lines report the intrinsic inhibition of the primary and secondary sources respectively and the grey areas represent the 90% confidence intervals.

Having established the most probable model architecture, we then compared the 16 models of time-dependent changes in intrinsic and extrinsic connectivities. One model (model 11) failed to converge during model inversion and was excluded from subsequent analysis. The pooled evidences of the remaining 15 models are shown in Fig. 6a.

The winning model (model 12) allowed changes in intrinsic connectivity in both the primary and the secondary sources. This model had greater evidence than any competing model. Typically, a difference in log evidence of three is considered strong evidence in favour of one model over another (this corresponds to a log marginal likelihood ratio of about 20 to 1). The difference between the best and next best models was much greater than three. Note that the model with the highest evidence was not the model with the greatest number of parameters (model 1). This reflects the complexity penalty inherent in Bayesian model comparison. In other words, changes in forward and backward connectivities did not improve accuracy sufficiently to justify their inclusion.

Finally we examined the posterior estimates (expectations) to quantify fluctuations in the parameters around seizure onset. The results are shown in Fig. 6b. Intrinsic connectivity increases markedly in both sources with seizure onset and then decreases within the first 20 s of seizure activity (the observed change in log scaling of about two corresponds to an eightfold increase in intrinsic connectivity). The trajectories are qualitatively consistent, given that they were estimated from independent data. The intrinsic connectivity modelled here is a sensitivity of (superficial) principal cells to presynaptic inputs from inhibitory interneurons. This fits comfortably with the conclusions of Wendling et al. (2005) who model seizure onset in terms of slow ensemble dynamics involving pyramidal cells and local interneurons, highlighting the increases in excitability that peak at seizure onset.

In summary, these results show that seizure onset appears to be mediated by an inhibition of superficial pyramidal cells in both sources. The key observation here is that the synaptic changes necessary to explain observed seizure activity (in terms of cross spectral density) are distributed, i.e. not restricted to the sole SOZ, and show slow dissociable time courses over several seconds. Furthermore, these changes are restricted to local or intrinsic fluctuations in synaptic parameters that are (presumably) a response to interactions among distal sources. Notice that the (reciprocal) extrinsic connections play a crucial role in the ensemble dynamics, in the sense that they mediate distributed interactions both before and after seizure onset. In short, the changes we have identified speak to a change in the recurrent interactions between excitatory principal cells (that originate forward type connections) and local inhibitory interneurons, reflecting a transient loss excitatory–inhibitory balance or gain control within a distributed epileptogenic network.

The reason that we can make definitive statements about directed connections among specific populations is that the (winning) DCM entails these specific changes. This illustrates the utility of having a biophysically explicit and plausible model of how data are caused — and the importance of Bayesian model comparison in adjudicating among different hypotheses.

## Discussion

Neuronal models are being increasingly used to characterise brain activity in different states, and the transition between these states. These transitions are most evident and crucial when the phenomenon to be modelled is the onset of an epileptic seizure.

A neuronal model of activity during different stages preceding and following seizure onset was proposed (Wendling et al., 2005), highlighting that the transition from the pre-ictal to the ictal state may not only be due to an increase of excitation (and a decrease of an inhibition) but rather to slow ensemble dynamics involving pyramidal cells and local interneurons, highlighting their increases in excitability that peak at seizure onset. A recent study (Nevado-Holgado et al., 2012) characterised the evolution of an absence seizure as a path through the parameter space of a neural mass model. In another approach (Hocepied et al., 2013) a similar scheme was proposed for early seizure detection. In both cases, the authors suggest that tracking a set of parameters over time can disclose the nature of ictogenesis. Characterising the trajectory of biophysical neural model parameters during seizure onset may provide insights into the underlying slow metabolic mechanisms.

The common theme in studies modelling seizure generation is a departure from the normal regime of functioning in populations of cells. This departure appears to be based on the interactions among excitatory pyramidal cells (Thomson and Radpour, 1991; Whittington et al., 1997) and their inhibitory interneurons (Miles et al., 1996; Banks et al., 1998; White et al., 2000). Several studies have investigated and reviewed the intracellular and extracellular mechanisms underlying slow changes in synaptic parameters during seizure activity (Jefferys et al., n.d.; McNamara, 1994, 1995; Isomura et al., 2008). McCormick and Contreras (2001) reported how periods of excitation, followed by synaptic inhibition and/or activation of intrinsic hyperpolarizing conductances can give rise to inter-ictal spikes, which can then be sustained during seizure activity.

Both David et al. (2008b) and Krishnan et al. (2013) addressed the causes of pathological synchronization, pointing out that changes in the extracellular ionic concentrations or modifications to excitation and inhibition can contribute to synchronized epileptiform firing. Increase in extracellular K^+^ concentration and decrease in Ca^2 +^ are the most likely candidates for mediating these slow changes in excitability (and disinhibition). Other variables related to energy metabolism (levels of extracellular K^+^, oxygen, ATP consumption) have been modelled as a slow *permittivity* variable in a dynamical model of seizure generation (Jirsa et al., 2014). This model highlights the separation of temporal scales in the genesis of seizure activity and highlights the role of slow fluctuations in excitability that our results appear to be consistent with.

Dynamical causal modelling was applied to intracranial EEG data recorded during 1 Hz electrical stimulation in patients with drug-resistant focal epilepsy (David et al., 2008b). DCM was used to model short term plasticity — as modulations of synaptic efficacies in either intrinsic or extrinsic connections. The observed fast transition from the pre-ictal to the ictal state may be due to changes in intrinsic connectivity. DCM revealed variations of the postsynaptic efficacies at the ictal zone. Their results suggested that electrically induced seizures in the temporal lobe could depend in part on a pre-ictal increase in sensitivity to hippocampal afferents from the temporal pole. Again, this is consistent with the notion that seizure activity results from distributed ensemble dynamics engaging both intrinsic and extrinsic connections.

It is clear that (slow) drifts in synaptic efficacy or coupling provide a sufficient account for the (fast) neuronal dynamics characteristic of seizure activity — and that these drifts involve involving regions distributed beyond the seizure onset zone. This perspective has been recently exploited. A bifurcation analysis of a physiological model of large-scale brain activity was used to obtain a parsimonious and unifying explanation of the defining features of seizure onset and spreading in Breakspear et al. (2006). Goodfellow et al. (2011) associated the emergency of epileptiform rhythms to two different scales of inhibition in a cortical neural mass model; in the work mentioned above: Jirsa et al. (2014) propose a minimal canonical model of epileptogenesis based upon a careful bifurcation analysis. This model exhibits spontaneous transitions between multi-stable states — resting on both slow and fast state variables. The dynamics emerging from both studies may provide a formal framework to study the neurophysiological mechanisms considered above.

In this paper we adopt a similar if complementary approach. We start from a canonical microcircuit model of neuronal sources and infer the evolution of its synaptic parameters around seizure onset. However, dynamic causal modelling takes its constraints from the known anatomy and physiology of neuronal circuits — as opposed to the formal (phenomenological) constraints offered by bifurcation analyses and dynamical systems theory. This means that the agenda is to parameterise seizure activity in terms of underlying synaptic mechanisms as opposed to their mathematical architecture. Crucially, we do not model a single epileptogenic region, but consider the distributed interactions with another population. This allowed us to use Bayesian model comparison to ask whether seizure activity was sufficiently explained by changes in one (epileptogenic) source — or required distributed changes throughout a simple network. Our results clearly point to a distributed explanation that rests upon coupled dynamics over both space and time. Nonetheless, given that the pathophysiology of epilepsy may be local (and mediated by non-specific extracellular factors), intrinsic plasticity may play a predominant role in seizure onset. In principle, it should be possible to extend this dynamic causal modelling approach to identify the causal architecture of these changes by explicitly modelling a slow (hidden) permittivity variable (such as extracellular potassium concentration) and testing different models. An important aspect of the current results is the dissociation in the temporal evolution of extrinsic (negligible) and intrinsic (marked) synaptic parameters. The nature of this dissociation may be important for understanding the intracellular and extracellular pathophysiology (what causes what) and clearly motivates further study in this area.

As with all dynamic causal modelling, the qualities of the models (model evidence) are only defined in relation to each other — and there is no supposition that the selected model represents some true or veridical architecture generating the data. In this sense, model comparison – and the interpretation of posterior estimates – is better thought of as testing specific hypotheses. In this instance, we wanted to test the hypothesis that a small number of (intrinsic) coupling strengths were sufficient to explain fluctuations in cross spectral density associated with seizure onset. To test more detailed hypotheses, one would have to specify a greater range of competing models and evaluate their evidence. A key point here is (as noted above) that at some point, the data at hand will not be able to disambiguate between models that are too complex (because their evidence will fall). It is at this point that one might turn to alternative sources of data — such as laminar-specific intracranial recordings.

In this paper we have focused on modelling spectral responses over epochs or windows around seizure onset using dynamic causal modelling for cross spectral density. It is interesting to consider alternative approaches. The first choice that one has to make in this context is whether to model the first-order responses in time or the second-order (spectral) responses in frequency space. In modelling endogenous activity, of the sort presented by seizure activity, modelling the time series can be difficult. This is because the time varying neuronal states generating data are unknown and have to be estimated. Although this is possible, it can be inefficient because one has to estimate both hidden neuronal states and unknown (connectivity) parameters. There are generalized (variational) Bayesian filtering techniques – that generalize the Kalman filter – which have been applied to fMRI time series (Li et al., 2011); however, they are relatively less common in electrophysiological time series analysis, see Freestone et al. (2011) for an application in the framework of neural field modelling. This is because the number of time bins and hidden neuronal states can be prohibitively large. In short, the more efficient way to model seizure activity is to focus on the time–frequency responses that reflect second-order statistics of neuronal activity. This means that hidden neuronal states do not have to be estimated and the data can be used to estimate unknown parameters (e.g., transfer functions and cross spectral predictions). In principle, it should be possible to model time varying parameters causing time-dependent changes in cross spectral measurements; however, we have chosen the simpler approach of using a piecewise linear approximation to these slow parameter changes. This allows us to use established model procedures for modelling complex cross spectra. We hope to compare this approach to explicit models of time frequency responses and, possibly, stochastic DCMs that estimate hidden neuronal states in the future.

This study is not meant to be a comprehensive illustration of dynamic causal modelling of seizure activity — rather a demonstration of the issues that are entailed and the nature of the questions that can be asked. The particular Bayesian updating scheme introduced here could be applied to measure synaptic modification on the scale of seconds to minutes. This may be useful for both epilepsy research and also studies of synaptic plasticity in studies of short or long-term potentiation or associative learning.

## Figures and Tables

**Table 1:** Model parameters used for subsequent dynamic causal modelling. The left column lists the parameters (corresponding to the equations in Fig. 3). The final two columns provide the prior mean and variance for dynamic causal modelling. Note that the variance is not the prior variance of the value per se but on its log scaling.

Description of parameter	Prior mean	Prior variance of log scaling
Intrinsic connections *d*_*ij*_ (Hz)	$\left[\frac{4}{5},\ldots ,\frac{1}{5}\right]\cdot 1000$	$\frac{1}{8}$
Extrinsic connections (Hz)	$\frac{1}{5}\cdot 1000$	$\frac{1}{8}$
Rate constants *κ*_*i*_ (Hz)	$\left[\frac{1}{2},\frac{1}{2},\frac{1}{16},\frac{1}{28}\right]\cdot 1000$	$\frac{1}{16}$
Slope of sigmoid *γ*	$\frac{2}{3}$	$\frac{1}{32}$
Intrinsic delays *τ* (ms)	1	$\frac{1}{32}$
Extrinsic delays *τ* (ms)	8	$\frac{1}{32}$
Amplitude of endogenous neuronal input	1	$\frac{1}{128}$
Power law exponent of neuronal input	1	$\frac{1}{128}$
Amplitude of measurement noise	1	$\frac{1}{128}$
Power law exponent of measurement noise	1	$\frac{1}{128}$
